# Medically unexplained symptoms and the risk of loss of labor market participation - a prospective study in the Danish population

**DOI:** 10.1186/s12889-015-2177-4

**Published:** 2015-09-02

**Authors:** Katja Loengaard, Jakob Bue Bjorner, Per Klausen Fink, Hermann Burr, Reiner Rugulies

**Affiliations:** National Research Centre for the Working Environment, Lerso Parkalle 105, DK-2100 Copenhagen, Denmark; Department of Public Health, University of Copenhagen, Copenhagen, Denmark; QualityMetric, Lincoln, RI USA; The Research Clinic for Functional Disorders and Psychosomatics, Aarhus University Hospital, Aarhus, Denmark; Federal Institute for Occupational Safety and Health (BAuA), Berlin, Germany; Department of Psychology, University of Copenhagen, Copenhagen, Denmark

## Abstract

**Background:**

Medically Unexplained Symptoms (MUS) are frequently encountered in general practice. However, little is known whether MUS affects labor market participation. We investigated the prospective association between MUS at baseline and risk of long-term sickness absence (LTSA), unemployment, and disability pensioning in a 5-year-follow-up study.

**Methods:**

In the Danish Work Environment Cohort Study 2005, 8187 randomly selected employees from the Danish general population answered a questionnaire on work and health. Responses were linked with national registers on prescribed medication and hospital treatment. Participants were classified with MUS if they: a) had reported three or more symptoms during the last month, and b) did not have a chronic condition, neither in the self-reported nor the register data. We assessed LTSA, unemployment, and disability pensioning by linking our data with National registers of social transfer payments.

**Results:**

Of the 8187 participants, 272 (3.3 %) were categorized with MUS. Compared to healthy participants, participants with MUS had an increased risk of LTSA (Rate ratio (RR) = 1.76, 95 % CI = 1.28–2.42), and of unemployment (RR = 1.48, 95 % CI = 1.02–2.15) during follow-up. MUS participants also showed an elevated RR with regard to risk of disability pensioning, however this association was not statistically significant (RR = 2.06, 95 % CI = 0.77–5.52).

**Conclusion:**

MUS seem to have a negative effect on labor market participation defined by LTSA and unemployment, whereas it is more uncertain whether MUS affects risk of disability pensioning.

## Background

Medically unexplained symptoms (MUS) name the phenomena of patients reporting diffuse physical symptoms of uncertain etiology, which cannot be conclusively attributed to any detectable physical disorders. Due to missing consensus concerning definition [1, 2], MUS is known also by other terms, such as somatization [3, 4], functional somatic symptoms [5, 6], and subjective health complaints [7].

MUS is one of the most common reasons for seeking health care [8–10]. The most often reported symptoms comprise headache, joint-, back- and abdominal pain [8, 11, 12]. The symptoms may vary in duration and in severity, ranging from mild symptoms to serious conditions with great suffering and disablement [2, 13–16]. Patients with MUS often report a severe impact on quality of life and have high health care utilization [10, 17, 18]. Further, patients with MUS often undergo unnecessarily medical examinations and treatments [19], which are costly in personal and economic terms [10, 17, 18, 20], both at a societal [21, 22] and at an individual level [17, 20, 23].

Case studies from medical practice suggest that MUS is a cause of sickness absence and reduced work function [24–27]. However, to our knowledge, no epidemiologic study has yet analyzed whether and to what extent MUS is a risk factor for long-term sickness absence (LTSA) and low labor market participation. Studies that have analyzed the association between number of symptoms and LTSA have for most part not taken into account whether the symptoms might be due to a medical condition. Instead, information about MUS have either been self-reported [28] or estimated by long-term sickness absence periods [26]. A Dutch study, for example, found that employees with a high symptom score accounted for approximately 40 % of work days lost during two-year follow-up [28]. A Danish study showed that the number of subjective health complaints predicted risk of sickness absence [29] when adjusting for self-reported history of seven diseases.

In this study, we aim to examine the association of MUS with LTSA, unemployment and disability pensioning in a representative sample of the Danish workforce. Using participants with no chronic disease and low symptom score as the reference group, we examine the risk of these three outcomes for a) participants without a diagnosed chronic disease but with a high symptom score, who we regard as possible MUS cases, b) participants with a chronic disease and a low symptom score, and c) participants with a chronic disease and a high symptom score. Further, we examine whether there is an interaction between presence/absence of chronic disease and high/low symptom score on risk of sickness absence, unemployment and disability pensioning.

## Methods

### Study design and population

This is a prospective cohort study that merges survey data with register data. Participants were drawn from the Danish Work Environment Cohort Study (DWECS) from 2005. DWECS was established in 1990, and consists of a random sample of Danish adult residents, drawn from the Central Population Register [30]. In 2005, 12,432 (62.5 %) Danish residents responded to the survey either by self-administered questionnaire provided on paper (*n* = 9271), on the internet (*n* = 448) or by telephone interview (*n* = 2713). For the purpose of analysis for this article, we excluded the 2713 participants who had responded on the telephone, because earlier analyses in DWECS had shown systematical differences in response patterns in the telephone interview survey compared to the paper and internet survey [31]. We further excluded 1525 participants who were 60 years or older, as we wanted to focus on participants of working age at baseline (18–59), before the possibility of early retirement. Finally, we excluded 7 participants with missing values on variables included in the analyses, yielding a final study population of 8187.

Using the unique social security number of the participants, we linked the survey data to data from four national registries: 1) The Danish Register-based Evaluation of Marginalization, (DREAM) [32, 33], that includes all social transfer payments made in Denmark since 1991; 2) The National Patient Registry [33] (NPR) that includes ICD codes for all somatic in-patient and out-patient hospital treatment in Danish hospitals since 1977; 3) The Danish Psychiatric Central Research Register [34] (PCRR) that includes ICD codes for all psychiatric in-patient and out-patient hospital treatment in Danish hospitals since 1969; and 4) The Danish National Prescription Registry [35] (DNPR) that includes information on prescribed drugs since 1995.

### Definition of participants with MUS and the three comparison groups

We categorized each participant according to high or low health symptom score. Symptoms were measured with the MUS-population-based scale (MUS-POP), an instrument that we had developed for studying symptoms scores in the DWECS sample. A detailed description of the development of the MUS-POP scale has been presented elsewhere (Loengaard, unpublished Ph.D. dissertation, can be requested from the corresponding author). Briefly, we used 28 self-reported health symptoms from the DWECS questionnaire and applied confirmatory factor analysis and Receiver Operating Characteristic (ROC) analysis to develop a scale that identifies respondents with health complaints not attributable to a known well-defined medical diagnosis, i.e., cases of MUS. We tested the True Positive Rate and the False Positive Rate of the scale with both poor self-rated health and doctor-diagnosed MUS. The analyses showed that 13 of the 28 items reflected specific symptom clusters rather than a global scale and we consequently excluded these items, leaving 15 items left for the MUS-POP scale with a good fit for a global scale. The 15 items were 1) Heat flush; 2) Palpitation; 3) Stomach pain; 4) Heartburn or reflux; 5) Bloated stomach; 6) Heavy head; 7) Muscle tension; 8) Hand, arm or elbow pain; 9) Dizziness; 10) Concentration problems; 11) Tight chest; 12) Itching, dry and irritated eyes; 13) Discomfort or nausea; 14) Throat irritation, voice fatigue or throat tension; and 15) No appetite. We found that a symptom score of 3 or higher on the MUS-POP global scale predicated poor self-reported health and doctor-diagnosed MUS (Loengaard, unpublished Ph.D. dissertation).

We further categorized each participant with regard to presence or absence of chronic disease. For this classification we combined information from self-report, information from NPR and PCRR registers on hospitalization with ICD-diagnose indicative of chronic disease, (e.g., allergy, asthma, coronary heart disease, cancer), and information from the DNPR register on prescription medication specific to chronic disease (e.g., insulin, asthma medication). Non-chronic diseases, for example influenza or pneumonia, non-psychotic psychiatric disorders, and diseases considered as part of functional somatic syndromes, such as migraine or fibromyalgia, were not included as indicators of chronic disease.

**Fig. 1:**
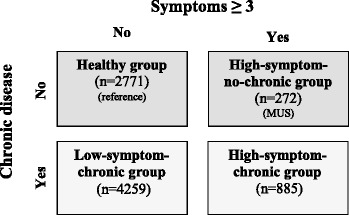
Categorization of the four compared groups according to symptom score and chronic disease

Based on the categorization with regard to low or high symptom scores and presence or absence of chronic disease we assigned each participant to one of four groups (Fig. 1): 1) low symptom score (<3 symptoms) and no chronic disease (denoted as the ‘healthy group’); 2) high symptom score (≥3 symptoms) and no chronic disease (denoted as the ‘high-symptom-no-chronic group’) who we regarded as possible MUS cases; 3) low symptom score (<3 symptoms) and chronic disease (denoted as the ‘low-symptom-chronic group’); and 4) high symptom score (≥3 symptoms) and chronic disease (denoted as the ‘high-symptom-chronic group’).

### Measurement of labor market status, sickness absence, unemployment and disability pensioning at baseline

In the DWECS survey, participants were first asked about their general labor market position, e.g., whether they were self-employed, manual workers, or non-manual workers or whether they were unemployed, studying, sick-listed or retired. Next, participants who were active in the labor market were asked about the specific type of work they were doing and the specific type of industry they were working in. Based on this information, we categorized participants into 12 groups, 8 groups of participants who were active in the labor market and 4 groups that were non-active (see Table 1 for details).

**Table 1:** Sociodemographic characteristics of the study sample, stratified by the four study groups

	Healthy group	High-symptom- no-chronic group (MUS)	Low-symptom-chronic group	High-symptom-chronic group	Chi^2^-test	Chi^2^-test	Chi^2^-test
	(*n* = 2771)	(*n* = 272)	(*n* = 4259)	(*n* = 885)	Healthy vs. High-symptom- no-chronic	Healthy vs. Low-symptom-chronic	Healthy vs. High-symptom-chronic
	% (n)	% (n)	% (n)	% (n)			
Sex							
Women	49.8 (1379)	74.6 (203)	55.9 (2382)	69.4 (614)			
Men	50.2 (1392)	25.4 (69)	44.1 (1877)	30.6 (271)	*p* < 0.0001	*p* < 0.0001	*p* < 0.0001
Age							
18–29	21.8 (605)	22.8 (62)	16.8 (715)	15.8 (140)			
30–39	25.0 (693)	19.5 (53)	24.1 (1027)	18.9 (167)			
40–49	28.0 (776)	27.6 (75)	27.8 (1185)	25.2 (223)			
50–59	25.2 (697)	30.2 (82)	31.3 (1332)	40.1 (355)	*p* = 0.1319	*p* < 0.0001	*p* < 0.0001
Education							
No vocational education	20.7 (553)	28.9 (75)	20.4 (836)	27.6 (235)			
Short education	14.2 (378)	13.9 (36)	14.0 (574)	17.1 (146)			
Medium length education	52.4 (1398)	48.1 (125)	52.2 (2140)	47.8 (408)			
Long education	12.7 (340)	9.2 (24)	13.5 (552)	7.5 (64)	*p* = 0.0147	*p* = 0.8566	*p* < 0.0001
Labor market position							
Self-employed	6.6 (181)	4.8 (13)	6.4 (271)	2.9 (26)			
Non-manual, high grade	13.9 (383)	6.6 (18)	13.5 (570)	6.3 (56)			
Non-manual, intermediate grade	15.4 (425)	15.1 (41)	17.1 (726)	11.2 (99)			
Non-manual, low grade	17.9 (494)	21.0 (57)	17.5 (129)	14.6 (129)			
Manual, skilled	15.2 (418)	13.7 (37)	12.0 (509)	10.2 (90)			
Manual, semi-/unskilled	12.1 (332)	15.5 (42)	9.6 (407)	10.3 (91)			
Work on special terms	0.5 (14)	1.5 (4)	2.2 (94)	5.9 (52)			
Vocational training/student	10.9 (301)	10.3 (28)	9.0 (382)	7.8 (69)			
Out of work, on leave	3.3 (92)	3.0 (8)	3.4 (145)	2.9 (26)			
Out of work, sickness absence	0.3 (9)	1.9 (5)	1.5 (62)	4.3 (38)			
Out of work, unemployed	3.2 (87)	4.8 (13)	4.1 (173)	6.8 (60)			
Out of work, retired	0.5 (14)	1.9 (5)	3.7 (156)	16.7 (147)	*p* < 0.0001	*p* < 0.0001	*p* < 0.0001

Sickness absence in DWECS was assessed by asking the participants “How many working days with sickness absence did you had during the last year?”. Responses were categorized into “low sickness absence” (0 to 5 days, i.e., no more than one working week during the last year) and “high sickness absence” (≥6 days). We also assessed LTSA in the DREAM register. DREAM includes information on all sickness absence compensations paid by Danish municipalities [32]. This compensation is paid by the municipalities to the employers, or, if a person is not employed, directly to the sick-listed person. For the purpose of analysis, we regarded sickness absence benefits of 8 weeks or more in the 12 months preceding the DWECS survey as indicative of LTSA. This cut-off point was chosen, because after 8 weeks of sickness absence the Danish municipalities will evaluate the status of the sick-listed person and will initiate return to work measures, if possible [36].

### Measurement of long-term sickness absence, unemployment and disability pensioning during follow-up

To examine risk of sickness absence and diminished labor market participation prospectively, we followed up all participants for 5 years in the DREAM register. During the follow-up period, we retrived from DREAM information on LTSA, i.e., sickness absence of 8 weeks or more, onset of unemployment of at 8 weeks or more, and disability pensioning.

### Measurement of covariates

As covariates, we assessed in DWECS sex, age, and education that was categorized into “no vocational education”, “short education”, “medium length education” and “long education”.

### Statistical analysis

We used chi^2^ tests to compare the sociodemographic characteristics (sex, age, education and labor market position) of the ‘healthy group’ with the ‘high-symptom-no-chronic group’, the ‘low-symptom-chronic group’, and the ‘high-symptom-chronic-group’ at baseline. Next, using logistic regression analyses we calculated odds ratios (OR) examining whether the ‘high-symptom-no-chronic group’ and the two chronic disease groups differed from the ‘healthy group’ with regard to being out of work at baseline and with regard to history of sickness absence during the 12 months preceding baseline. Analyses were performed with and without adjustment for sex, age, and education. Analyses for being out of work at baseline were carried out for all participants, whereas analyses for self-reported and register-based sickness absence were carried out only for participants who were in work. We also analyzed whether there was an interaction effect of chronic disease (yes/no) with symptom score (high/low) on being out of work and on sickness absence.

Using Poisson regression we calculated rate ratios (RR) comparing participants in the ‘high-symptom-no-chronic group’ and the two chronic disease groups with participants in the ‘healthy group’ regarding risk of LTSA, unemployment and disability pensioning during the 5-year follow-up period. Only participants who were active at the labor market at baseline were included in these analyses. In the analyses on sickness absence, we excluded participants with registered sickness absence of one month before or after baseline. In the analyses on unemployment, we excluded participants with registered unemployment one month before or after baseline. We calculated the RR both for each of the three endpoints, separately and for the three endpoints combined. We also analyzed whether there was an interaction effect of chronic disease (yes/no) with symptom score (high/low) on the three endpoints and the combination of the three endpoints.

In all analyses participants were followed-up until event, old-age pension, emigration out of Denmark, death or end of follow-up, whichever came first. In the analyses on sickness absence and unemployment, participants were further censored on the day of disability pensioning and women were censored for maternity leave. All analyses were adjusted for sex, age, and education at baseline. The analyses on risk of sickness absence were further adjusted for sickness absence of at least eight weeks during the 12 months before baseline, and the analyses on risk of unemployment were further adjusted for unemployment of at least eight weeks during the 12 months before baseline. All analyses were conducted in SAS, version 9.3.

In addition to the main analyses, we conducted two sensitivity analyses. In the first sensitivity analysis we excluded participants aged 55–59, because in this age range sickness absence or unemployment may be used by some workers as an ‘exit pathway’ bridging the time until early retirement benefits become available. In the second sensitivity analysis we re-defined chronic disease by excluding three self-reported conditions (‘hay fever’, ‘skin disease’ and ‘allergy’) that we considered as less disabling than the other diseases.

This study has been approved by the acquired Danish Data Protection Agency, journal number: 2011-54-1148, in order of creating a register of personal data. According to Danish legislation, this study did not need approval by an ethic committee, because it was neither a clinical trial nor did it include biological material.

## Results

### Study sample characteristics

Of the 8187 participants, 2771 (33.8 %) were categorized into the ‘healthy group’, 272 (3.3 %) into the ‘high-symptom-no-chronic group’, 4259 (52.0 %) into the ‘low-symptom-chronic group’, and 885 (10.8 %) into the ‘high-symptom-chronic group’.

Table 1 shows the sociodemographic characteristics of the four groups at baseline. The ‘high-symptom-no-chronic group’ differed in several characteristics from the participants in the ‘healthy group’ and the ‘low-symptom-chronic group’. Participants in the ‘high-symptom-no-chronic group’ were more likely to be women (74.6 vs. 49.8 and 55.9 %), and to have no vocational education (28.9 vs. 20.7 and 20.4 %), and were less likely to be non-manual workers of high occupational grade (6.6 vs. 13.9 and 13.5 %). Further, retirement was more likely in the ‘high-symptoms-no-chronic group’ than in the ‘healthy group’ (1.9 vs. 0.5 %) but less likely than in the ‘low-symptom-chronic group’ (3.7 %) and the ‘high-symptom-chronic group’ (16.7 %).

### Being out of work at baseline and sickness absence before baseline

Table 2 shows analyses on being out of work at baseline and on LTSA in the 12 months before baseline. Compared to the ‘healthy group’, the ‘high-symptom-no-chronic group’ showed a higher likelihood of being out of work in the crude analysis, but no significant difference in the adjusted analysis (OR = 1.35, 95 % CI = 0.89–2.06). In contrast, the groups with chronic disease -- with and without high symptoms -- had significantly increased odds ratios of being out of work at baseline. In separate analyses, we evaluated a possible interaction between reporting symptoms and having a chronic disease with regards to being out of work. We found a statistically significant interaction (*P* = 0.0037), indicating that people with both a high symptom level and a chronic disease had a higher likelihood of being out of work than would have been predicted from each of the two indicators alone.

**Table 2:** Comparison of labor market participation and sickness absence at baseline in the four groups

	Number	Cases % (N)	Crude OR (95 % CI)	Adjusted OR (95 % CI)
Out of work at baseline				
Healthy group	2771	7.40 (205)	1 (Reference)	1 (Reference)
High-symptom-no-chronic group (MUS)	272	11.40 (31)	**1.61 (1.08–2.40)**	1.35 (0.89–2.06)
Low-symptom-chronic group	4259	12.63 (538)	**1.81 (1.53–2.14)**	**1.71 (1.43–2.04)**
High-symptom-chronic group	885	30.62 (271)	**5.53 (4.52–6.76)**	**4.52 (3.65–5.60)**
Six days or more of sickness absence, 12 months before baseline (self-reported)				
Healthy group	2566	17.42 (447)	1 (Reference)	1 (Reference)
High-symptom-no-chronic group (MUS)	241	31.54 (76)	**2.18 (1.63–2.92)**	**2.16 (1.61–2.92)**
Low-symptom-chronic group	3721	23.68 (881)	**1.47 (1.30–1.67)**	**1.47 (1.29–1.67)**
High-symptom-chronic group	614	29.83 (256)	**3.39 (2.80–4.10)**	**3.28 (2.70–3.99)**
Long-term sickness absence of 8 consecutive weeks, 12 months before baseline (register data)				
Healthy group	2771	2.06 (57)	1 (Reference)	1 (Reference)
High-symptom-no-chronic group (MUS)	272	7.72 (21)	**3.64 (2.00–6.62)**	**3.36 (1.83–6.16)**
Low-symptom-chronic group	4259	5.56 (237)	**2.37 (1.70–3.30)**	**2.29 (1.63–3.21)**
High-symptom-chronic group	885	12.88 (114)	**6.94 (4.72–10.19)**	**6.20 (4.18–9.19)**

Out of work analyses: All participants (*n* = 8187); Sickness absence analyses: Only participants who were actively participating in the labor market at baseline (*n* = 7142)

Adjusted OR are adjusted for sex, age, education

Odds ratios not including unity are printed in **bold**

Participants in the ‘high-symptom-no-chronic group’ were more likely to have self-reported sickness absence of 6 days or more (OR = 2.16, 95 % CI = 1.61–2.92) in the adjusted analyses than the ‘healthy group’. This odds ratio was higher than the risk for the chronic disease group with a low symptom score (OR = 1.47, 95 % CI = 1.29–1.67), but lower than the risk for the chronic disease group with a high symptom score (OR = 3.28, 95 % CI = 2.70–3.99). In separate analyses, we found no interaction between symptom level and chronic disease on likelihood of sickness absence before baseline. Analyses, of register-based LTSA of 8 consecutive weeks or more, showed similar results. The odds ratio was significantly increased for the ‘high-symptom-no-chronic group’ (OR = 3.36, 95 % CI = 1.83–6.16), higher than the odds ratio in the ‘low-symptom-chronic group’ and lower than the odds ratio in the ‘high-symptoms-chronic group’. Similarly, we found no indications of an interaction.

### Risk of long-term sickness absence, unemployment and disability pensioning during follow-up

Table 3 shows the prospective associations with risk of LTSA, unemployment and disability pensioning among those participants from the four groups who were actively participating in the labor market at baseline. Compared to the ‘healthy group’, participants in the ‘high-symptoms-no-chronic group’ were at increased risk of LTSA in the adjusted analyses (RR = 1.76, 95 % CI = 1.28–2.42). Similar to results for the retrospective analyses, the risk in the ‘high-symptom-no-chronic group’ was higher than the risk in the ‘low-symptom-chronic group’ and lower than the risk in the ‘high-symptom-chronic group’.

**Table 3:** Risk of long-term sickness absence, unemployment and disability pensioning during 5-year follow-up in the four groups

	Number	Cases % (N)	Crude RR (95 % CI)	Adjusted RR (95 % CI)
Long-term sickness absence 8 weeks				
Healthy group	2433	10.81 (263)	1 (Reference)	1 (Reference)
High-symptom-no-chronic group (MUS)	220	21.36 (47)	**2.09 (1.53–2.85)**	**1.76 (1.28–2.42)**
Low-symptom-chronic group	3433	15.38 (528)	**1.46 (1.26–1.69)**	**1.38 (1.19–1.61)**
High-symptom-chronic group	519	30.44 (158)	**3.12 (2.56–3.08)**	**2.57 (2.09–3.15)**
Unemployment 8 weeks				
Healthy group	2431	10.32 (251)	1 (Reference)	1 (Reference)
High-symptom-no-chronic group (MUS)	224	14.73 (33)	**1.47 (1.02–2.11)**	**1.48 (1.02–2.15)**
Low-symptom-chronic group	3471	11.38 (395)	1.11 (0.94–1.30)	1.14 (0.97–1.34)
High-symptom-chronic group	560	20.89 (117)	**2.12 (1.70–2.62)**	**2.05 (1.64–2.58)**
Disability pensioning				
Healthy group	2756	0.80 (22)	1 (Reference)	1 (Reference)
High-symptom-no-chronic group (MUS)	267	2.25 (6)	**2.82 (1.14–6.96)**	2.06 (0.77–5.52)
Low-symptom-chronic group	4097	3.05 (125)	**3.86 (2.46–6.08)**	**3.59 (2.23–5.77)**
High-symptom-chronic group	738	11.38 (84)	**14.93 (9.34–23.88)**	**12.32 (7.51–20.21)**
Any of the three events				
Healthy Group	2367	18.50 (438)	1 (Reference)	1 (Reference)
High-symptom-no-chronic group (MUS)	211	30.81 (65)	**1.78 (1.37–2.31)**	**1.56 (1.19–2.04)**
Low-symptom-chronic group	3294	23.53 (775)	**1.29 (1.15–1.46)**	**1.27 (1.13–1.43)**
High-symptom-chronic group	488	43.85 (214)	**2.65 (2.25–3.13)**	**2.43 (2.05–2.87)**

All analyses restricted to participants with labor market participation at baseline (sample, *n* = 7858)

Long-term sickness absence: adjusted for sex, age, education, and history of long-term sickness absence. Participants on sickness absence at baseline or at one month preceding or following baseline were excluded (sample, *n* = 6605)

Unemployment: adjusted for sex, age, education, and history of unemployment. Participants on unemployment at baseline or at one month preceding or following baseline were excluded (sample, *n* = 6686)

Disability pensioning: adjusted for sex, age, education

Any of the three events: Adjusted for sex, age, education, history of long-term sickness absence, history of unemployment. Participants on sickness absence or unemployment at baseline or at one month preceding or following baseline were excluded (sample, *n* = 6360)

Rate Ratios not including unity are printed in **bold**

The ‘high-symptoms-no-chronic group’ showed an increased risk of unemployment in the adjusted analyses (RR = 1.48, 95 % CI = 1.02–2.15). There was also a significantly increased risk in the ‘high-symptoms-chronic group’ but not in the ‘low-symptom-chronic group’.

Participants in the ‘high-symptom-no-chronic group’ showed an increased risk of disability pensioning in the crude analysis (RR = 2.82, 95 % CI = 1.14–6.96). After adjustment for covariates the RR dropped to 2.06 and statistical significance was lost. The risk of disability pensioning was significantly increased for both the’low-symptom-chronic group’ (RR = 3.59) and the ‘high-symptom-chronic group’ (RR = 12.32).

In none of the prospective analyses did we find a statistically significant interaction between level of symptoms and chronic disease.

When we combined all three events, LTSA, unemployment and disability pensioning, we found that the participants in the ‘high-symptoms-no-chronic group’ had a significantly increased risk of experiencing at least one of the three events during follow-up compared to the participants in the ‘healthy group’ (RR = 1.56, 95 % CI = 1.19–2.04). This risk was higher than the risk for the ‘low-symptom-chronic group’ but lower than the risk for the ‘high-symptom-chronic group’. We did not find a significant interaction effect in the analysis.

### Sensitivity analyses

We conducted two sensitivity analyses. First, we repeated the prospective analyses on risk of LTSA, unemployment and disability pension (Table 3) while excluding the 1294 participants who were 55 years or older. Compared to the ‘healthy group’, the ‘high-symptom-no-chronic group’ had a RR of 1.90 (95 % CI: 1.37–2.63) for LSTA and 1.58 (95 % CI: 1.08–2.32) for unemployment in this sensitivity analysis, similar to the results from the main analysis reported in Table 3. For disability pensioning the RR increased more markedly when excluding participants aged 55 years or older and was statistically significant (RR = 3.06, 95 % CI: 1.08–8.62), whereas it was not statistically significant in the main analysis (Table 3).

Second, we repeated the analyses from Table 3, while no longer considering self-reported ‘hay fever’, ‘skin disease’ and ‘allergy’ as a chronic disease, because these conditions presumably have a relatively low impact on disability. Using this new chronic disease definition, the RR for the ‘high-symptom-no chronic group’ was 2.11 (95 % CI: 1.60–2.68) for LTSA, 1.64 (95 % CI: 1.24–2.18) for unemployment and 3.29 (95 % CI: 1.67–6.47) for disability pensioning. Thus, also in this second sensitivity analysis, the most substantial change was observed for disability pensioning. For the two chronic disease groups, the RR for disability pensioning was 4. 27 (95 % CI: 3.13–7.13, ‘low-symptom-chronic’) and 14.43 (95 % CI: 9.42–22.34, ‘high-symptom-chronic’), respectively, when using the re-defined chronic disease measure.

## Discussion

### Summary of results

In this study with a random sample of the Danish population, we found that compared to healthy participants, participants in the ‘high-symptom-no-chronic group’, which we regarded as possible MUS cases, were more often female, had lower education, were less likely to be non-manual workers of high occupational grade and more likely to be retired. Participants in this group had an increased risk of LTSA and unemployment during the five-year follow-up. They also had an increased risk of disability pensioning that was statistically significant in the crude analysis of the main sample and in both the crude and adjusted analyses of two sensitivity analyses, although not in the adjusted analysis of the main sample. For both LTSA and unemployment, the risk in the ‘high-symptom-no-chronic group’ was higher than among participants with a low symptom score and a chronic disease, but lower than among participants with a high symptom score and a chronic disease.

The baseline analyses showed that participants from the two chronic disease groups and (in the crude analysis) also participants from the ‘high-symptom-no-chronic group’ were more likely to be out of work than the ‘healthy group’. It could have been assumed that participants who, despite their chronic disease or their high symptom score, were working at baseline, had somehow found a way to reconcile their health condition with job requirements and therefore would not be at increased risk of labor market participation during follow-up. This was not the case. The ‘high-symptom-non-chronic group’ and the ‘high-symptom-chronic group’ were at increased risk of unemployment and both chronic groups were at increased risk of disability pensioning. Thus, although our sample in the prospective analysis consisted of employees who had managed to keep labor market attachment at baseline despite their health condition, they were still at increased risk of losing labor market attachment during follow-up.

### Comparison with previous findings

Previous studies have found that high symptoms scores were associated with sickness absence [29, 37], and that people with high symptom scores among sick-listed were more disabled, had prolonged sick leaves and were more often dismissed than people with a low symptom score [26]. Additionally, a study in the Netherlands showed that those people reporting most symptoms accounted for most parts of the work days lost during a two-year follow-up [28]. Although these studies were not exactly comparable with our study, their findings match our findings about increased risk of LTSA.

It has been well agreed on that MUS can be disabling [12, 13, 38], and that LTSA increases the risk of both unemployment and disability pensioning [39, 40]. However, to the best of our knowledge there have not been any population-based studies examining the risk of unemployment and disability pensioning among people with MUS. The findings from our study suggest that people with MUS are at increased risk for unemployment and maybe also for disability pensioning, although statistical significance was lost in the adjusted analysis. Further, our results showed that participants with a high symptom score and a chronic disease had a 12-fold increased risk for disability pensioning, which was substantially higher than in any other of the examined groups.

### Methodological consideration

This is a non-experimental, observational epidemiological study and therefore causal inference has to be drawn with caution as we cannot rule out that unmeasured factors may have confounded the association of our predictor variables and the different endpoints. This said, we believe that our study has several methodological strengths that support a causal interpretation of the findings. These strengths are in particular the use of a representative sample of a national workforce, the fairly large sample and the decent response rate of 62.5 %. The availability of Danish national registers allowed us to assess prevalence of a diagnosed disease not only by self-report but also by registered ICD-codes. In the prospective analyses, the endpoints of LTSA, unemployment and disability pensioning were assessed by another national register, which allowed a complete follow-up of all participants.

The main methodological challenge of this study is the assessment of MUS. We had no clinical examinations available in the study, and therefore our measurement of MUS was based on combining a high symptom score with the absence of a doctor-diagnosed chronic disease, which we assessed both by self-report and register-data. We reasoned that people who are suffering from a high symptom score and at the same time do not have a doctor-diagnosed chronic disease are likely to suffer from symptoms that cannot be medically explained, i.e., from MUS. In an earlier analysis, this method was able to predict doctor-diagnosed MUS (Loengaard, unpublished Ph.D. thesis).

We acknowledge, though, that it is likely that this assessment method has led to some misclassification. Because we had only information available about chronic disease before and until baseline, we cannot rule out that for some participants the symptoms were medically unexplained at baseline, but got a medical explanation at some point during follow-up. Thus, while such participants were correctly classified at baseline, they might not be correctly classified during the whole follow-up.

On the other hand, it is possible that some of the high symptom scores in the chronic disease group were not caused by the specific chronic disease of the participants but had other causes, including causes that were not medically explained – i.e., there might had been in some cases a co-existing of both chronic disease and MUS. Consequently, these participants should have been regarded as the MUS participants.

In our study almost two thirds of the participants had a chronic disease, which is a very high number taken into account that these participants were mostly part of the workforce. This high prevalence may be due to our inclusive approach that combined both self-reported and register-based doctor diagnosed diseases and which included also diseases that had their onset many years ago. Thus, a certain amount of the chronic diseases, in particular those that we assessed via the registers, might not had played a role in the daily life of the participants when they filled in the survey.

Finally, while the initial response rate of 62.5 % is decent for a large-scale epidemiological study, we decided to exclude all participants who had responded to the questions in a telephone interview instead of filling in the paper questionnaire or responding to an internet questionnaire. This reduced our sample size markedly and has reduced statistical power in the analyses. We would have preferred to include all participants, however, we decided against this because of previous evidence from DWECS showing that response patterns to several key questions deviated substantially depending on whether data was collected by a telephone interview or by paper questionnaire or internet survey [31].

### Implication

It is well-accepted that MUS severely affects quality of life [38, 41] and predicts impaired health status [42]. Our results show that MUS also puts employees at a considerable risk of LTSA and unemployment. LTSA in itself is known to have negative consequences [43] at a societal level by increasing the cost of social benefit payment, for the workplace due to productivity loss, and for the individual where LTSA increases the risk of adverse economic and social conditions [44–46]. Studies have shown that employees on LTSA often feel guilty for not being able to work and for being a burden for society [45]. This feeling of guilt may cause alienation and withdrawal from social activities [45]. Some studies have suggested that these feelings of guilt and alienation and the withdrawal from social activities are even more enhanced in employees’ sick listed with MUS, as the lack of a medical diagnosis and the need to’proof’ [47] that the symptoms are’real’ may act as an additional psychological strain [47]. Such strain may result into passivity and social isolation [24], which could initiate a process of marginalization [39].

Our results are potentially important for health social security professionals who are working with people suffering from MUS. These professionals need to be aware of that employees with MUS are at increased risk of LTSA, unemployment and maybe also disability pensioning, even though employees with MUS lack a medical diagnosis. Health and social security professionals may consider informing employees with MUS about this increased risk. Social security professionals may further consider including employees with MUS into prevention or re-integration programs aimed at individuals at high risk for loss of labor market participation.

## Conclusions

To conclude, our findings suggest that MUS not only affects well-being, but have severe consequences in terms of LTSA and unemployment. We recommend that these possible consequences are taken into consideration by health and social security professionals who are working with people suffering from MUS.

## Abbreviations

MUS: Medically Unexplained Symptoms
MUS-POP: Medically Unexplained Symptoms Population symptom scale
LTSA: Long-term Sickness Absence
DWECS: Danish Work Environment Cohort Study
NPR: National Patient Register
PCRR: The Danish Psychiatric Central Research Register
DNPR: The Danish National Prescription Register
DREAM: The Danish Register-based Evaluation and Marginalization
ROC: Receiver Operating Characteristic

## References

[1] McFarlane AC, Ellis N, Barton C, Browne D, Van HM (2008). The conundrum of medically unexplained symptoms: questions to consider. Psychosomatics.

[2] Sharpe M, Carson A (2001). “Unexplained” somatic symptoms, functional syndromes, and somatization: do we need a paradigm shift?. Ann Intern Med.

[3] Brown TM (2004). Somatization. Medicine.

[4] Mayou R (1993). Somatization. Psychother Psychosom.

[5] Barsky AJ, Borus JF (1999). Functional somatic syndromes. Ann Intern Med.

[6] Mayou R, Farmer A (2002). ABC of psychological medicine - functional somatic symptoms and syndromes. Br Med J.

[7] Eriksen HR, Ihlebaek C, Ursin H (1999). A scoring system for subjective health complaints (SHC). Scand J Public Health.

[8] Cherry DK, Woodwell DA, Rechtsteiner EA (2007). National Ambulatory Medical Care Survey: 2005 summary. Adv Data.

[9] Fink P, Rosendal M (2008). Recent developments in the understanding and management of functional somatic symptoms in primary care. Curr Opin Psychiatry.

[10] Tomenson B, McBeth J, Chew-Graham CA, MacFarlane G, Davies I, Jackson J, Littlewood A, Creed FH (2012). Somatization and health anxiety as predictors of health care use. Psychosom Med.

[11] Eriksen HR, Ursin H (2004). Subjective health complaints, sensitization, and sustained cognitive activation (stress). J Psychosom Res.

[12] Hiller W, Rief W, Brahler E (2006). Somatization in the population: from mild bodily misperceptions to disabling symptoms. Soc Psychiatry Psychiatr Epidemiol.

[13] Harris AM, Orav EJ, Bates DW, Barsky AJ (2009). Somatization increases disability independent of comorbidity. J Gen Intern Med.

[14] Jackson JL, Kroenke K (2006). Managing somatization: medically unexplained should not mean medically ignored. J Gen Intern Med.

[15] Kroenke K, Price RK (1993). Symptoms in the community. Prevalence, classification, and psychiatric comorbidity. Arch Intern Med.

[16] Whitehead LC (2006). Quest, chaos and restitution: living with chronic fatigue syndrome/myalgic encephalomyelitis. Soc Sci Med.

[17] Barsky AJ, Orav EJ, Bates DW (2005). Somatization increases medical utilization and costs independent of psychiatric and medical comorbidity. Arch Gen Psychiatry.

[18] Fink P (1992). The use of hospitalizations by persistent somatizing patients. Psychol Med.

[19] Fink P. Surgery and medical-treatment in persistent somatizing patients. J Psychosom Res. 1992;36(5):439–47. 10.1016/0022-3999(92)90004-l 1535658

[20] Reid S, Crayford T, Patel A, Wessely S, Hotopf M (2003). Frequent attenders in secondary care: a 3-year follow-up study of patients with medically unexplained symptoms. Psychol Med.

[21] Konnopka A, Schaefert R, Heinrich S, Kaufmann C, Luppa M, Herzog W, et al. Economics of medically unexplained symptoms: a systematic review of the literature. Psychother Psychosom. 2012;81(5):265–75. 10.1159/000337349 22832397

[22] Konnopka A, Kaufmann C, König HH, Heider D, Wild B, Szecsenyi J, et al. Association of costs with somatic symptom severity in patients with medically unexplained symptoms. J Psychosom Res. 2013;75(4):370–5. 10.1016/j.jpsychores.2013.08.011 24119945

[23] Hansen MS, Fink P, Sondergaard L, Frydenberg M (2005). Mental illness and health care use: a study among new neurological patients. Gen Hosp Psychiatry.

[24] Aamland A, Malterud K, Werner EL (2012). Phenomena associated with sick leave among primary care patients with medically unexplained physical symptoms: a systematic review. Scand J Prim Health Care.

[25] Al-Windi A (2005). The influence of complaint symptoms on health care utilisation, medicine use, and sickness absence: a comparison between retrospective and prospective utilisation. J Psychosom Res.

[26] Hoedeman R, Blankenstein AH, Krol B, Koopmans PC, Groothoff JW (2010). The contribution of high levels of somatic symptom severity to sickness absence duration, disability and discharge. J Occup Rehabil.

[27] Kroenke K, Spitzer RL, Williams JB (2002). The PHQ-15: validity of a new measure for evaluating the severity of somatic symptoms. Psychosom Med.

[28] Roelen CAM, Koopmans PC, Groothoff JW (2010). Subjective health complaints in relation to sickness absence. Work.

[29] Poulsen OM, Persson R, Kristiansen J, Andersen LL, Villadsen E, Ørbæk P. Distribution of subjective health complaints, and their association with register based sickness absence in the Danish working population. Scand J Public Health. 2013;41(2):150–7. 10.1177/1403494812471909 23287396

[30] Feveile H, Olesen O, Burr H, Bach E (2007). Danish work environment cohort study 2005: from idea to sample design. Stat Transit-New Ser.

[31] Feveile H, Olsen O, Hogh A (2007). A randomized trial of mailed questionnaires versus telephone interviews: response patterns in a survey. BMC Med Res Methodol.

[32] Burr H, Pedersen J, Hansen JV (2011). Work environment as predictor of long-term sickness absence: linkage of self-reported DWECS data with the DREAM register. Scand J Public Health.

[33] Hjollund NH, Larsen FB, Andersen JH (2007). Register-based follow-up of social benefits and other transfer payments: accuracy and degree of completeness in a Danish interdepartmental administrative database compared with a population-based survey. Scand J Public Health.

[34] Mors O, Perto GP, Mortensen PB (2011). The Danish psychiatric central research register. Scand J Public Health.

[35] Kildemoes HW, Sorensen HT, Hallas J (2011). The Danish national prescription registry. Scand J Public Health.

[36] Labriola M, Christensen KB, Lund T, Nielsen ML, Diderichsen F (2006). Multilevel analysis of workplace and individual risk factors for long-term sickness absence. J Occup Environ Med.

[37] Ihlebaek C, Brage S, Eriksen HR (2007). Health complaints and sickness absence in Norway, 1996–2003. Occup Med (Lond).

[38] Hoedeman R, Krol B, Blankenstein N, Koopmans PC, Groothoff JW (2009). Severe MUPS in a sick-listed population: a cross-sectional study on prevalence, recognition, psychiatric co-morbidity and impairment. BMC Public Health.

[39] Hultin H, Lindholm C, Moller J (2012). Is there an association between long-term sick leave and disability pension and unemployment beyond the effect of health status?--a cohort study. PLoS One.

[40] Lamberg T, Virtanen P, Vahtera J, Luukkaala T, Koskenvuo M (2010). Unemployment, depressiveness and disability retirement: a follow-up study of the Finnish HeSSup population sample. Soc Psychiatry Psychiatr Epidemiol.

[41] Chaturvedi SK, Desai G, Shaligram D (2006). Somatoform disorders, somatization and abnormal illness behaviour. Int Rev Psychiatry.

[42] Creed FH, Tomenson B, Chew-Graham C, Macfarlane GJ, Davies I, Jackson J, et al. Multiple somatic symptoms predict impaired health status in functional somatic syndromes. Int J Behav Med. 2013;20(2):194–205. 10.1007/s12529-012-9257-y 22932928

[43] Sieurin L, Josephson M, Vingaard E (2009). Positive and negative consequences of sick leave for the individual, with special focus on part-time sick leave. Scand J Public Health.

[44] Bryngelson A (2009). Long-term sickness absence and social exclusion. Scand J Public Health.

[45] Floderus B, Alexanderson K, Aronsson G (2005). Self-estimated life situation in patients on long-term sick leave. J Rehabil Med.

[46] Hoedeman R, Blankenstein AH, Koopmans PC, Groothoff JW (2013). What bothers the sick-listed employee with severe MUPS?. Scand J Public Health.

[47] Werner A, Malterud K (2003). It is hard work behaving as a credible patient: encounters between women with chronic pain and their doctors. Soc Sci Med.

